# Effect of arabinoxylan on colonic bacterial metabolites and mucosal barrier in high‐fat diet‐induced rats

**DOI:** 10.1002/fsn3.1164

**Published:** 2019-08-12

**Authors:** Shanshan Li, Yanan Sun, Xinxin Hu, Wen Qin, Cheng Li, Yuntao Liu, Aiping Liu, Ye Zhao, Dingtao Wu, Derong Lin, Qing Zhang, Daiwen Chen, Hong Chen

**Affiliations:** ^1^ College of Food Science Sichuan Agricultural University Yaan Sichuan China; ^2^ College of Animal Science and Technology Sichuan Agricultural University Chengdu Sichuan China; ^3^ Institute of Animal Nutrition Sichuan Agricultural University Chengdu Sichuan China

**Keywords:** Arabinoxylan, gene expression, microbial metabolites, mucosal barrier

## Abstract

The aim of this study was to demonstrate the effect of arabinoxylan on colonic mucosal barrier and metabolomic profiles in high‐fat diet‐induced rats. A total of 20 six‐week‐old male rats were arranged randomly to two groups (*n* = 10/group), including a high‐fat diet (HFD) group and a high‐fat supplemented with arabinoxylan diet (AXD) group. Results showed that feeding AXD reduced serum lipopolysaccharide in rats after 5 weeks. In colonic digesta, *Escherichia coli* population was reduced, while *Lactobacillus*, *Bifidobacterium,* and *Bacteroidetes* populations were increased in AXD group. Metabolomics assay found that the different abundances of 84 metabolites were observed, involving lipid, carbohydrate, and nitrogenous metabolism in colonic digesta. In colonic mucosa, AXD up‐regulated gene level of tight‐junction‐related proteins. Meanwhile, lower TNF‐α and IL‐1β levels were related to TLR4/NF‐κB/MyD88 pathway in AXD group. In conclusion, arabinoxylan could change colonic microbial metabolism and improve the colonic mucosal barrier via modulating intestinal microflora and tight junction proteins.

**High lights**

- Arabinoxylan reduced* E. coli* and increased *Lactobacillus* and *Bifidobacterium* populations in rats.
- Arabinoxylan changed colonic metabolomics profiles in rats.
- Arabinoxylan improved colonic mucosal barrier function in rats.

## 1 INTRODUCTION

Currently, continuous consumption of excessive fat is considered to increase the risk of metabolic disorder in animals’ model and humankinds’ (Riccardi, Giacco, & Rivellese, 2004), while the high‐fat diet‐induced metabolic disorder probably resulted from the variation in intestinal microorganisms and the damage of intestinal mucosal barrier (Moreira, Texeira, Ferreira, Peluzio, & Alfenas, 2012). Therefore, the prebiotics improving intestinal microenvironment are considered as a candidate to interfere high‐fat diet‐induced metabolic disorder.

Cereal nonstarch polysaccharide (NSP) was reported to have prebiotic potential as fermentable substrates of probiotics. Arabinoxylans (AX), constituting about 60% of NSP in cereal (about 9.5% of cereal), have received a lot more attention in the prebiotic functions (Grootaert et al., 2009). In gut, AX was degraded by microbial enzymes and stimulated the proliferation of probiotics as carbon source (Broekaert et al., 2011). AX (supplemented with 5.6% in high‐fat diet) more gradually fermented along the colon exerting important physiologic functions, including modulation of mucin degradation, change in fermentation, and regulation of intestinal bacteria (Van et al., 2011). Besides, AX (6% in high‐fat diet) supplementation in the diet was demonstrated to counteract high‐fat diet‐induced intestinal dysbiosis together with an improvement of obesity (Neyrinck et al., 2011). Periodical intake of AX brought about a more advantageous fermentative profile in overweight and obesity (Salden et al., 2017). There are many papers on the study of how gut microbiota influences host metabolites and mucosal barrier (Han et al., 2016). However, few studies paid attention to the effect of arabinoxylan on high‐fat diet‐induced systemic change in colonic mucosal barrier and microbial metabolites. Therefore, we used metabolomics technology to assay the change in intestinal metabolites and focused on the effects of arabinoxylan on colonic metabolic profiles and mucosal barrier in high‐fat diet‐induced rat in this study.

## 2 MATERIALS AND METHODS

The experimental protocol used in the present study was carried out in line with the Institutional Review Board (No. IRB14044) and the Institutional Animal Care and Use Committee of the Sichuan Agricultural University (No. DKY‐B20140302) (Zhang et al., 2019).

### 2.1 Experimental design

Arabinoxylan (85% purity) was purchased from Shanghai Ryon Biological Technology CO, Ltd. Twenty male Sprague Dawley rats (16‐week‐old) were purchased from Chengdu Dashuo Experimental animal Co., Ltd. Rats were fed ad libitum with 12‐hr daylight cycle. The animal house maintained at 22 ± 2°C with relative humidity between 50% and 60%. After one week of acclimatization, the rats were arranged completely randomly to two groups (*n* = 10/group), including a high‐fat diet (HFD) group and a high‐fat supplemented with AX diet (AXD) group. The composition of synthetic diets (HFD and AXD) is presented in Table S1, and AXD is constituted of 94% HFD and 6% AX. The lard as a fat source was supplemented, including 0.90 mg/g cholesterol, 38.2% oleic acid, 25.1% palmitic acid, 11.8% stearic acid, and 10.7% linoleic acid. The standard methods from Chinese Food Safety National Standard (GB 5009.5–2016) were used to measure the diet protein content. The standard methods from Chinese Food Safety National Standard (GB 5009.6–2016) were used to measure diet fat content. The standard methods from William (1995) were used to measure diet carbohydrate and energy content (Liu et al., 2018; Liu, Yan, Zhang, Hu, & Zhang, 2019a). The feed intake of rats was recorded every day, while body weight was documented per week during the trial period.

### 2.2 Sample collection

After 5 weeks, the eyeballs of rats were removed and collected blood samples in Eppendorf tubes. The blood samples were centrifuged at 3,500 *g* for 15 min at 4°C after 60‐min standing, and then, the serum was obtained. After killed by dislocating their cervical vertebra, the abdomens of rats were immediately opened to collect intestinal sections and content. The digesta of colon were collected, immediately quick‐frozen using liquid nitrogen, and transferred into −80°C freezer for further analysis. The segments (4 cm) at 90% of the length of the small intestine (90% SI) and colon were put in 10% formaldehyde buffer for morphology analysis. The mucosal scrapings were collected from 90% SI and colon segments (10 cm), which were immediately quick‐frozen using liquid nitrogen and transferred into −80°C freezer for further analysis.

### 2.3 Blood biochemical parameters assay

The contents of total bile acids (TBA), triglycerides (TG), total cholesterol (TC), high‐density lipoprotein cholesterol (HDL‐c), and low‐density lipoprotein cholesterol (LDL‐c) in serum were analyzed using commercial assay kits (Nanjing Jiancheng Bioengineering Institute). Meanwhile, lipopolysaccharide (LPS), tumor necrosis factor‐α (TNF‐α), interleukin‐1β (IL‐1β), and interleukin‐6 (IL‐6) contents were measured using an anti‐rat ELISA kit (Bethyl Laboratories. Inc.).

### 2.4 The number of goblet cell assay

The samples of 90% SI and colon (5 cm) were put in 10% neutral buffered formalin and embedded using paraffin. The samples embedded in paraffin were sliced into sections (4 mm) using a microtome. The slices were stained with periodic acid schiff and counter‐stained with hematoxylin. At least, ten complete villi or crypts per intestinal site were chosen to determine goblet cell number per villus or crypt.

### 2.5 Assessment of secretory IgA (slgA) level, intestinal alkaline phosphatase (IAP) activity, and the parameters of inflammation response in 90% SI and colonic mucosa

After suspended in 9 ml PBS, the colonic mucosal scrapings (1 ml) were homogenized using ultrasonic homogenizer. The homogenates were centrifugation at 2,500 *g* for 15 min, and then, the supernatant was collected and centrifuged at 12,000 *g* for 5 min. Then, the Bradford brilliant blue method was used to analyze the total protein content of the samples. While, anti‐rat ELISA kits (Bethyl Laboratories. Inc.) were used to measure sIgA, TNF‐α, IL‐1β, and IL‐6 concentration. The alkaline phosphatase assay kit (ABACOM) was used to measure IAP activity.

### 2.6 Gene expression assayed using RT‐PCR technology in 90% SI and colon

TRIzol reagent (Invitrogen) was used to extract the total RNA of intestinal mucosal scrapings following its manufacturer's instructions. The purity and content of RNA were determined spectrophotometrically (Beckman Coulter DU800; Beckman Coulter Inc.) (Liu, Zhang, Li, Yan, & Zhang, 2019b). Then, the RNA samples met the requirements were reverse‐transcribed into complementary DNA with the PrimeScript RT reagent kit (Takara). RT‐PCR for quantification analysis of target genes was performed on the Opticon DNA Engine (Bio‐Rad) with SYBR Green PCR reagents (Takara). Glyceraldehyde‐3‐phosphate dehydrogenase (GAPDH) (GenBank NM001206359) and β‐actin (GenBank DQ452569) were selected as the housekeeping gene. The primers of β‐actin, GAPDH, NF‐κB, MyD88, claudin‐1, ZO‐1, Occludin, Bax, and Bcl‐2 were synthesized commercially by Life Technologies Limited **(**Table S2). The cycle profile consisted of denaturation at 95°C for 2 min, followed by 40 cycles of 95°C for 20 s, 63°C for 30 s, and 72°C for 60 s. The PCR products were then analyzed for homogeneity by melting curve analysis. The housekeeping genes did not vary between diets (*p* = .81) in duplicates. Each sample and standard were moved simultaneously in duplicate on the same PCR plate, and the average of each duplicate copy was used for statistical analysis.

### 2.7 Quantitative RT‐PCR quantification of 90% SI and colonic bacteria

Microorganisms DNA were extracted from the intestinal content with the Stool DNA Kit (Omega Bio‐Tek) (Yan, Zhang, Guo, Zhang, & Liu, 2019). Group‐specific primers of *Escherichia coli*, *Bifidobacterium*, *Lactobacillus*, and *Bacteroidetes* based on 16S rDNA sequences PCR assay were synthesized commercially by Life Technologies Limited (Table S3). The reaction protocol consisted of one cycle of predenaturation at 95°C for 2 min; 50 cycles of denaturation at 95°C for 15 s; annealing at 60°C for 30 s; and extension at 72°C for 50 s. The volume system of 20 ml was run in each reaction. In the same run, each test was carried out in duplicate. The cycle threshold of each sample was compared with a standard curve made by diluting genomic DNA as 10‐fold serial dilution from cultures. The Neubauer hemocytometer was used to confirm cell counted before DNA extraction. In order to confirm the specificity and sensitivity of the assays, the PCR assays were determined with a set of intestinal microorganisms’ species as controls.

### 2.8 Metabolomics assay

Samples of colonic content were prepared for gas chromatography–mass spectrometry (GC‐MS) analysis following previous studies (Gao et al., 2009; Polakof et al., 2013). Colonic content (0.3 g) was mixed with 0.3 ml of ultrapure water and then were ultracentrifuged at 4°C and 12,000*g* for 2 hr. Then, supernatants were collected and derivatized. Each 1‐μL aliquot of the derivatives was injected into a mass spectrometric detector system (Agilent Technologies). Helium as a carrier gas passed through the column at a constant flow rate. Agilent “retention time locking” was used to control the precision of retention time (RT), where phenylalanine was chosen as the calibrated compound. For CI mode, the same capillary column and GC‐MS parameters were set. Pure methane was chosen as reagent gas.

Multivariate data assay was carried out on the normalized GC‐MS datasets using the SIMCA14 software package (Umetrics). The resulted three‐dimensional data were fed to SIMCA14 software package, which were performed principal component analysis (PCA) and orthogonal projections to latent structures discriminant analysis (OPLS‐DA). Nevertheless, the loading plot is complicated due to many variables. In order to refine this analysis, the first principal component of variable importance projection (VIP) was acquired. Then, the VIP values higher than 1.0 were first chosen as different metabolites. At last, the surplus variables were analyzed by Student's *t* test (*p* > .05), and variables were abandoned between two diet groups.

### 2.9 Statistical analysis

For metabolomics analysis, univariate and multivariate statistics were carried out on the data matrix. The other data were subjected to unpaired *t* tests to determine differences between HFD and AXD groups with SPSS 21.0 software (SPSS Inc.). The α‐level used for significance was 0.05. Results were expressed as the mean ± standard deviation (*SD*).

## 3 RESULTS AND DISCUSSION

### 3.1 The body weight, serum lipid metabolic, and immunity parameters

There is lower daily weight gain in AXD group than HFD group, as a result, a reduction in final body weight of AXD group was observed relative to HFD group (Table 1). Additionally, rats fed AXD had lower serum LDL‐c and TG level relative to HFD. Lower serum LPS and TNF‐α concentration were observed in AXD than HFD group.

**Table 1:** The body weight, serum lipid metabolic, and immunity parameters in rats

Item	HFD	AXD
Initial weight, g	220 ± 9.78	220 ± 9.77
Final body weight, g	422 ± 27.4^a^	394 ± 19.5^b^
Daily weight gain, g/day	6.11 ± 0.77^a^	5.26 ± 0.47^b^
Daily food intake, g/day	23.5 ± 1.72	22.3 ± 0.59
In serum		
TG, mmol/L	1.95 ± 0.58^a^	1.02 ± 0.17^b^
TC, mmol/L	2.09 ± 0.29	1.86 ± 0.18
HDL‐c, mmol/L	0.65 ± 0.07	0.74 ± 0.08
LDL‐c, mmol/L	0.23 ± 0.05^a^	0.19 ± 0.06^b^
TBA, μmol/L	4.80 ± 0.61	4.65 ± 0.60
LPS, ng/ml	1.35 ± 0.08^a^	0.89 ± 0.12^b^
TNF‐α, ng/L	69.8 ± 5.60^a^	55.8 ± 5.40^b^
IL−1β, ng/L	62.5 ± 7.3	61.3 ± 6.18
IL−6, ng/L	48.3 ± 6.12	43.3 ± 5.77

Note

Values (means ± *SD*, *n* = 10) with different letters within a row are significantly different (*p* < .05).

Abbreviations: AXD, a high‐fat supplemented with arabinoxylan diet; HDL‐c, high‐density lipoprotein cholesterol; HFD, a high‐fat diet; IL‐1β, interleukin‐1β; IL‐6, interleukin‐6; LDL‐c, low‐density lipoprotein cholesterol; LPS, lipopolysaccharide; TBA, total bile acid; TC, total cholesterol; TG, triglyceride; TNF‐α, tumor necrosis factor‐α.

The final body weight in rats fed AXD was reduced, which demonstrated that AX could have an advantageous impact on improving high‐fat diet‐induced overweight. Excessive lipid intake could result in higher blood TG and LDL‐c level and increase the risk of dyslipidemia (Parikh, Joshi, Menon, & Shah, 2010). In this study, 6% AX extract was supplemented in high‐fat diet, which was considered as an appropriate dose to alleviate metabolic disorders caused by high‐fat diet was similar with previous studies reported by Van et al. (2011) and Neyrinck et al. (2012), which was considered as an appropriate dose to alleviate metabolic disorder caused by high‐fat diet. And this supplementation reduced the risk of dyslipidemia in HFD group via reducing blood TG and LDL‐c level in rats. Besides, metabolic syndrome such as obesity and dyslipidemia were reported to associate with systemic chronic low‐grade inflammation induced by increased blood LPS level (Cani et al., 2008). The study from Neyrinck et al. (2012) showed that wheat‐derived arabinoxylan oligosaccharides reduced blood LPS level in diet‐induced obese mice. In addition, blood LPS level was positively correlated with the change in intestinal microbiota and mucosal permeability (Cani et al., 2008). With serum LPS level lowering, arabinoxylan supplementation could inhibit inflammation response via reducing blood proinflammatory cytokines (TNF‐α) level, in line with the result from Salden et al. (2017) that a decreased blood TNF‐α level was observed in arabinoxylan supplemented diet.

### 3.2 Intestinal bacteria

**Figure 1:**
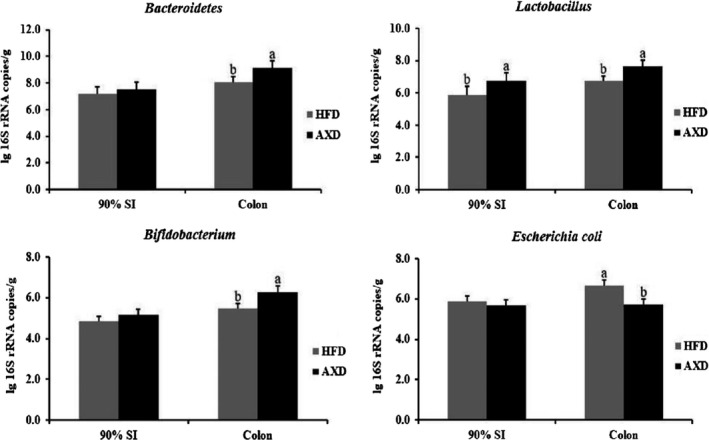
Effects of the high‐fat diet on intestinal bacteria in the colonic content. HFD: a high‐fat diet, AXD: a high‐fat supplemented with arabinoxylan diet. Values (means ± *SD*, *n* = 10) with different letters within the column are significant different (*p* < .05)

The number of colonic bacteria changed when rats were fed different diets (Figure 1). The population of *Lactobacillus* increased in AXD group relative to HFD group in 90% SI and colon. Correspondingly, higher *Bifidobacterium* and *Bacteroidetes*, and lower *Escherichia coli* populations were observed in rats fed AXD relative to HFD in colon. Wheat AX fractions were shown to provoke the proliferation of probiotics (*Bifidobacterium* and *Lactobacillus* species) (Chen et al., 2015; Laere, Hartemink, Bosveld, Schols, & Voragen, 2000), in accordance with our results in the AXD group. *Lactobacillus* acting as a probiotic has shown a positive role in animal by regulating microbial composition, intestinal development, and immune status (Liu et al., 2014). The previous study demonstrated that *Bacteroidetes* and *Bifidobacterium* are involved in the degradation of arabinoxylans in the gut (Broekaert et al., 2011). Besides, the establishment of *E. coli* in AXD group was reduced possibly by competitive exclusion with intestinal probiotics (Chen et al., 2013).

### 3.3 PCA and OPLS‐DA

**Figure 2:**
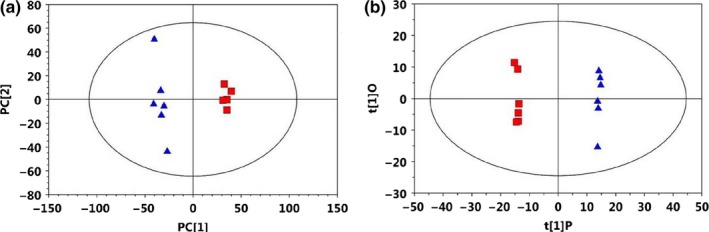
(a) PCA (*R* ^2^X = 0.39, *Q* ^2^ = 0.056) and (b) OPLS‐DA (*R* ^2^X = 0.376, *R* ^2^Y = 0.999, *Q* ^2^ = 0.916) score plots based on the ^1^H NMR spectra of metabolites in colonic digesta obtained from HFD (black circles) and AXD (red squares). Each symbol represents a mouse; ellipses represent 95% CIs on the basis of Hotelling's T^2^ statistic. OPLS‐DA model development used 5 mice in HFD group and 5 mice in AXD group; therefore, score plots represent results from the PLS‐DA model. PLS‐DA model validation was performed by using the remaining 5 mice/group. OPLS‐DA, orthogonal projections to latent structures discriminant analysis

**Figure 3:**
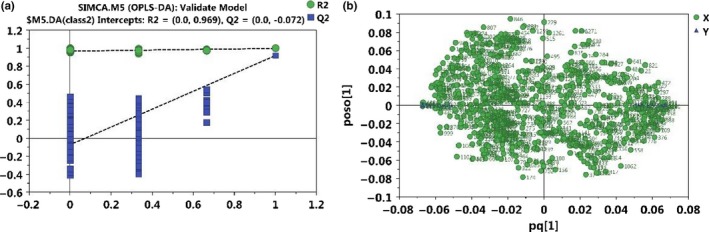
(a) Permutations test and (b) loading plot of OPLS‐DA model from HFD and AXD. The green line represents the regression line for *R* ^2^ (green circle) and the blue line for *Q* ^2^ (blue square)

PCA was initially carried out on the colonic digesta spectral data. PCA results showed that data in rats from the HFD and AXD group were basically separated in their metabolic profiles of colonic digesta (Figure 2a). Furthermore, the metabolic changes in the rats from the two groups were analyzed by using OPLS‐DA. Two hundred permutations were carried out, and R^2^ and Q^2^ values were plotted in Figure 3a. The low values of Q^2^ intercept indicated the robustness of the models, which showed a low risk of overfitting and reliability. On the basis of the OPLS‐DA, a loading plot was established to show the contribution of variables to difference between two groups (Figure 3b).

### 3.4 Differential metabolites in colonic content

A total of 750 metabolites were detected, 325 of which were annotated. The different abundances of 84 metabolites were observed (VIP values > 1.0 and *p* ≤ .05) (Table 2). In colon, 13 lipid metabolism‐related metabolites decreased in the AXD group relative to HFD group. Eleven carbohydrate metabolism‐related metabolites increased in the AXD group relative to HFD group. Eleven nitrogenous metabolism‐related metabolites increased, while xanthurenic acid and glycocyamine 3 contents decreased in the AXD group relative to HFD group.

**Table 2:** Differential metabolites in colonic content in rats

Metabolites	RT/min	*p*‐value	Fold change (AXD/HFD)
Lipids metabolism			
Citraconic acid 4	13.49	<.01	0.16
Lauric acid	17.30	<.01	<0.01
Myristic acid	19.46	<.05	0.60
Palmitoleic acid	21.22	<.05	0.53
Pelargonic acid	13.65	<.05	0.76
Pentadecanoic acid	20.45	<.01	0.38
Prostaglandin e2 1	27.93	<.01	0.07
Succinate semialdehyde 1	11.10	<.05	0.52
Prostaglandin a2 3	27.02	<.01	0.07
21‐hydroxypregnenolone 4	30.30	<.01	0.54
2‐methoxyestrone 1	28.62	<.01	<0.01
Androsterone 1	25.90	<.01	<0.01
Stigmasterol	32.00	<.01	0.19
Zymosterol 2	31.25	<.01	1.97
Carbohydrates metabolism			
1,5‐anhydroglucitol	15.45	<.01	7.58
Allose 1	17.07	<.01	8.97
D‐glyceric acid	13.16	<.01	2.34
Lactulose 1	26.93	<.01	8.87
Purine riboside	24.33	<.01	6.67
Threitol	15.40	<.01	5.64
Xylose 1	17.24	<.01	8.08
Nitrogenous metabolism			
ɤ‐aminobutyric acid	15.84	<.01	5.53
O‐Succinylhomoserine	21.35	<.01	3.72
Alpha‐Aminoadipic acid	17.89	<.01	9.36
Asparagine 5	16.37	<.01	2.01
Beta‐Alanine 2	14.54	<.01	2.63
L‐Allothreonine 1	13.93	<.01	1.63
L‐cysteine	16.12	<.01	9.67
Lysine	20.18	<.01	3.73
Picolinic acid	13.14	<.05	1.81
Pyrrole−2‐carboxylic acid	13.52	<.01	4.84
Serine 1	13.58	<.01	1.73
2'‐Deoxycytidine 5'‐triphosphate	19.52	<.05	8.02
Xanthurenic acid	23.42	<.05	<0.01
Glycocyamine 3	19.29	<.05	0.29

Note

HFD: a high‐fat diet, AXD: a high‐fat supplemented with arabinoxylan diet.

The previous evidence showed that gut microbiota influences the host metabolites in intestinal content (Martin et al., 2010). Our results showed that arabinoxylan supplementation in HFD changed colonic microbiota‐related lipid metabolism, carbohydrate metabolism, and nitrogenous metabolism. AXD raised zymosterol level, which is a precursor of cholesterol (Lange, Echevarria, & Steck, 1991) and reduced androsterone, 2‐methoxyestrone, and 21‐hydroxypregnenolone level synthesized by cholesterol as a precursor in colonic digesta (Chaudhuri & Anand, 2017), meaning that the intake of arabinoxylan changed colonic cholesterol metabolism. While, prostaglandin E2 and spermine were reported to be involved in bile acids metabolism (Mitsuharu et al., 2012). Xanthurenic acid, a product of tryptophan degradation, could induce cell pathological apoptosis via activation of cell caspases (Malina, Richter, Mehl, & Hess, 2001). The decrease in colonic xanthurenic acid level in AXD indicated that arabinoxylan could alleviate high‐fat diet‐induced cell pathological apoptosis, in line with the change in colonic cell apoptosis‐related gene (Bax and Bcl‐2) mRNA level. Previous studies found that 3,4‐dihydroxybenzoic acid improved rat digestive tract health by modulating oxidative DNA damage and inhibiting carcinoma cell growth (Guglielmi, Luceri, Giovannelli, Dolara, & Lodovici, 2003; Lin, Chen, Huang, & Wang, 2007). Aminoadipic acid was considered as a biomarker and therapy for diabetes to raise the risk of glucose‐related metabolic disorder (Wang et al., 2013). Hence, aminoadipic acid raised in AXD group demonstrated that arabinoxylan could have a potency to decrease the risk of metabolic disorder induced by high‐fat diet. In amino acids metabolism, o‐succinyl homoserine is an intermediate in the formation of cystathionine and consequently of methionine by *E. coli* (Rowbury & Woods, 1964). However, colonic cystathionine and methionine level did not raise with o‐succinyl homoserine level increasing in AXD group, possibly derived from *E. coli* abundance reduced by arabinoxylan supplementation in AXD. Besides, an increase in colonic glutamate, lysine, serine, cysteine, and asparagine content in AXD implied that arabinoxylan supplementation promoted biosynthesis of amino acid. Zhou et al. (2017) reported that serine relieved oxidative stress through providing methionine cycle and glutathione synthesis, mainly by condensing with homocysteine to synthesize cysteine. AXD influenced GABAergic synapse and increased colonic ɤ‐aminobutyric acid (GABA) level. Several studied reported that GABA could improve hypertension as the fermented product of intestinal lactic acid bacteria (Chang, Chiu, & Fu, 2016), in line with our result of *Lactobacillus* species abundance increased. Arabinoxylan supplementation reduced colonic epsilon‐caprolactam level as a low toxic substance, which could be degraded by several bacteria (Esikova, Akatova, & Taran, 2014). These results inferred that arabinoxylan could improve intestinal mucosal barrier via modulating colonic metabolites production.

### 3.5 Intestinal mucosal gene expression

**Figure 4:**
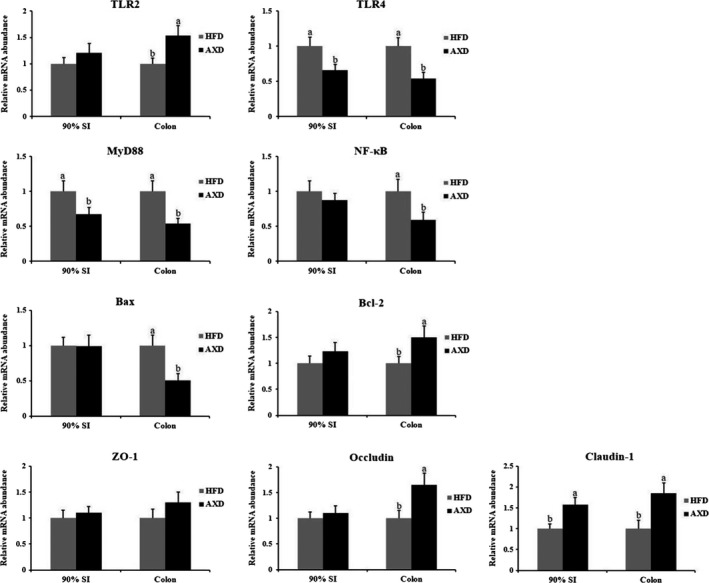
Effects of the high‐fat diet on gene expression in the colonic content. HFD: a high‐fat diet, AXD: a high‐fat supplemented with arabinoxylan diet. Values (means ± *SD*, *n* = 10) with different letters within the column are significant different (*p* < .05)

Intake of arabinoxylan influenced gene expression of colonic mucosal barrier‐related proteins in rats (Figure 4). At 90% SI, HFD supplemented arabinoxylan up‐regulated occludin mRNA abundance and down‐regulated TLR4 and MyD88 mRNA abundances in rats. In colon, claudin‐1 and TLR2 mRNA abundances were up‐regulated in AXD group relative to HFD group. While, lower TLR4, MyD88, and NF‐κB mRNA abundances were found in rats feeding AXD relative to HFD. For the cell apoptosis, Bax gene expression was down‐regulated, while Bcl‐2 gene expression was up‐regulated in rats fed AXD relative to HFD. Intercellular structures termed tight junctions were considered to regulate intestinal permeability. Tight junction proteins regulated intestinal permeability via preventing the paracellular diffusion of hazardous materials (e.g., intestinal LPS) across the mucosal epithelium (Ulluwishewa et al., 2011). AX supplemented in high‐fat diet up‐regulated the mRNA level of tight‐junction‐related proteins compared with those in HFD group (Neyrinck et al., 2011). Similar study found that AX supplementation up‐regulated gene expression of occludin and claudins (Salden et al., 2017). *Lactobacillus* improved the permeability of Caco‐2 cells via up‐regulating occludin and ZOs mRNA levels in vitro (Mccann et al., 2010). Similarly, *Bifidobacterium* prevented intestinal barrier disorder in mouse via maintaining the location and tight junction proteins expression (Bergmann et al., 2013). These results suggested that colonic gene expression level of tight junction proteins up‐regulated in AXD group in part originated from higher colonic probiotics (*Lactobacillus *and *Bifidobacterium*) populations in the present study. Meanwhile, Toll‐like receptor 2 (TLR2) was reported to preserve intestinal mucosal epithelial tight‐junction‐associated integrity via regulating ZO‐1 and claudins (Gibson et al., 2008). While, arabinoxylan could activate LPS receptor TLR 4 to modulate the immune response (Fadel et al., 2017). Lower MyD88 and NF‐κB mRNA abundance in AXD group implied that arabinoxylan supplementation in HFD could modulate intestinal permeability in colon via inhibiting TLRs/MyD88/NF‐κB pathway in the present study.

### 3.6 Intestinal goblet cell number and mucosal factors

**Figure 5:**
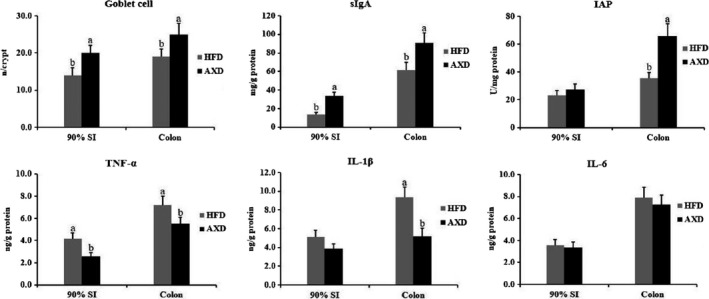
Effects of the high‐fat diet on sIgA, IAP, and inflammation response in the colon. HFD: a high‐fat diet, AXD: a high‐fat supplemented with arabinoxylan diet, sIgA: secretory IgA, IAP: intestinal alkaline phosphatase. Values (means ± *SD*, *n* = 10) with different letters within the column are significantly different (*p* < .05)

The number of goblet cell increased in crypt of 90% SI and colon when rats feeding AXD relative to HFD (Figure 5). As shown in Figure 3, HFD supplemented arabinoxylan decreased TNF‐α level and increased sIgA level at 90% SI and colon. Besides, higher colonic IAP activity was observed in rats feeding AXD than HFD. Arabinoxylan supplemented raised the number of goblet cell in colonic crypt of piglets (Chen et al., 2015), in agreement with our result in AXD group. SIgA was secreted in mucosal tissue to protect the intestinal mucosa by preventing the challenge from potentially commensal bacteria, foreign proteins, and infectious agents (Corthesy, 2013). In intestinal brush‐border, IAP may detoxify LPS and prevent bacterial invasion across the intestinal mucosal barrier in vitro and in vivo* (*Malo et al., 2010 *)*, partially leading to lower serum LPS content in AXD group along with IAP activity increasing in the present study. Meanwhile, the coincubation of *Lactobacillus* strain and Caco‐2 cells promoted IAP activity when Caco‐2 cells were challenged with the potent mycotoxin in vitro (Turner et al., 2008), in line with higher prebiotics in AXD group. Proinflammatory cytokines (TNF‐α and IL‐1) have been shown to dysregulate tight junction proteins via raising intestinal mucosal permeability (Capaldo & Nusrat, 2009). Besides, IL‐1β harmed intestinal mucosal barrier function in Caco‐2 (Al‐Sadi, Ye, Said, & Ma, 2010) and the TNF‐α raised intestinal mucosal epithelial permeability (Ma, Hoa, Akotia, & Chen, 2002). These results indicated that arabinoxylan could improve intestinal barrier function in HFD group via reducing intestinal mucosal proinflammatory cytokines level.

## 4 CONCLUSION

In summary, the results presented here indicate that feeding arabinoxylan could not only modulate intestinal bacteria, but also change intestinal microbial metabolism in high‐fat diet‐induced rats. Additionally, along with improvement of intestinal tight junction and immune barrier, arabinoxylan supplemented in HF reduced serum lipopolysaccharide level.

## CONFLICTS OF INTEREST

There is no conflict of interest associated with the authors of this paper.

## ETHICAL APPROVAL

There is no conflict of interest in this study. And the study has conformed to the Declaration of Helsinki, US. The experimental protocol used in the present study was carried out in line with the Institutional Review Board (No. IRB14044) and the Institutional Animal Care and Use Committee of the Sichuan Agricultural University (No. DKY‐B20140302).

## References

[fsn31164-bib-0001] Al‐Sadi, R. , Ye, D. , Said, H. M. , & Ma, T. Y. (2010). IL‐1β‐Induced Increase in Intestinal epithelial tight junction permeability is mediated by MEKK‐1 activation of canonical NF‐κB pathway. American Journal of Pathology, 177, 2310–2322. 10.2353/ajpath.2010.100371 21048223 PMC2966790

[fsn31164-bib-0002] Bergmann, K. R. , Liu, S. X. L. , Tian, R. , Kushnir, A. , Turner, J. R. , Li, H.‐L. , … De Plaen, I. G. (2013). Bifidobacteria stabilize claudins at tight junctions and prevent intestinal barrier dysfunction in mouse necrotizing enterocolitis. American Journal of Pathology, 182, 1595–1606. 10.1016/j.ajpath.2013.01.013 23470164 PMC3644725

[fsn31164-bib-0003] Broekaert, W. F. , Courtin, C. M. , Verbeke, K. , Van de Wiele, T. , Verstraete, W. , & Delcour, J. A. (2011). Prebiotic and other health‐related effects of cereal‐derived Arabinoxylans, Arabinoxylan‐Oligosaccharides, and Xylooligosaccharides. Critical Reviews in Food Science and Nutrition, 51, 178–194. 10.1080/10408390903044768 21328111

[fsn31164-bib-0004] Cani, P. D. , Bibiloni, R. , Knauf, C. , Waget, A. , Neyrinck, A. M. , Delzenne, N. M. , & Burcelin, R. (2008). Changes in gut microbiota control metabolic endotoxemia‐induced inflammation in high‐fat diet‐induced obesity and diabetes in mice. Diabetes, 57, 1470–1481. 10.2337/db07-1403 18305141

[fsn31164-bib-0005] Capaldo, C. T. , & Nusrat, A. (2009). Cytokine regulation of tight junctions. Biochimica Et Biophysica Acta, 1788, 864–871. 10.1016/j.bbamem.2008.08.027 18952050 PMC2699410

[fsn31164-bib-0006] Chang, V. H. , Chiu, T. H. , & Fu, S. C. (2016). In vitro anti‐inflammatory properties of fermented pepino (*Solanum muricatum*) milk by γ‐aminobutyric Acid‐producing *Lactobacillus brevis* and an in vivo animal model for evaluating its effects on hypertension. Journal of the Science of Food & Agriculture, 96, 192–198. 25582456 10.1002/jsfa.7081

[fsn31164-bib-0007] Chaudhuri, A. , & Anand, D. (2017). Cholesterol: Revisiting its fluorescent journey on 200th anniversary of Chevruel’s “cholesterine”. Biomedical Spectroscopy and Imaging, 6, 1–24. 10.3233/bsi-170166

[fsn31164-bib-0008] Chen, H. , Mao, X. , He, J. , Yu, B. , Huang, Z. , Yu, J. , … Chen, D. (2013). Dietary fibre affects intestinal mucosal barrier function and regulates intestinal bacteria in weaning piglets. British Journal of Nutrition, 110, 1837–1848. 10.1017/S0007114513001293 23656640

[fsn31164-bib-0009] Chen, H. , Wang, W. , Degroote, J. , Possemiers, S. , Chen, D. , De Smet, S. , & Michiels, J. (2015). Arabinoxylan in wheat is more responsible than cellulose for promoting the intestinal barrier function in weaned male piglets. Journal of Nutrition, 145, 51–58. 10.3945/jn.114.201772 25378684

[fsn31164-bib-0010] Corthesy, B. (2013). Multi‐faceted functions of secretory IgA at mucosal surfaces. Frontiers in Immunology, 4, 185 10.3389/fimmu.2013.00185 23874333 PMC3709412

[fsn31164-bib-0011] Esikova, T. Z. , Akatova, E. V. , & Taran, S. A. (2014). Bacteria that degrade low‐molecular linear epsilon ‐caprolactam oligomers. Applied Biochemistry & Microbiology, 50, 463–470. 10.1134/S0003683814050044 25707105

[fsn31164-bib-0012] Fadel, A. , Plunkett, A. , Li, W. , Tessu Gyamfi, V. E. , Nyaranga, R. R. , Fadel, F. , … Ashworth, J. J. (2017). Modulation of innate and adaptive immune responses by arabinoxylans. Journal of Food Biochemistry, 42(2), e12473 10.1111/jfbc.12473

[fsn31164-bib-0013] Gao, X. , Pujosguillot, E. , Martin, J. F. , Galan, P. , Juste, C. , Jia, W. , & Sebedio, J. L. (2009). Metabolite analysis of human fecal water by gas chromatography/mass spectrometry with ethyl chloroformate derivatization. Analytical Biochemistry, 393, 163 10.1016/j.ab.2009.06.036 19573517

[fsn31164-bib-0014] Gibson, D. L. , Ma, C. , Rosenberger, C. M. , Bergstrom, K. S. B. , Valdez, Y. , Huang, J. T. , … Vallance, B. A. (2008). Toll‐like receptor 2 plays a critical role in maintaining mucosal integrity during *Citrobacter rodentium*‐induced colitis. Cellular Microbiology, 10, 388–403. 10.1111/j.1462-5822.2007.01052.x 17910742

[fsn31164-bib-0015] Grootaert, C. , Van den Abbeele, P. , Marzorati, M. , Broekaert, W. F. , Courtin, C. M. , Delcour, J. A. , … Van de Wiele, T. (2009). Comparison of prebiotic effects of arabinoxylan oligosaccharides and inulin in a simulator of the human intestinal microbial ecosystem. FEMS Microbiology Ecology, 69, 231–242. 10.1111/j.1574-6941.2009.00712.x 19508502

[fsn31164-bib-0016] Guglielmi, F. , Luceri, C. , Giovannelli, L. , Dolara, P. , & Lodovici, M. (2003). Effect of 4‐coumaric and 3,4‐dihydroxybenzoic acid on oxidative DNA damage in rat colonic mucosa. British Journal of Nutrition, 89, 581–587. 10.1079/BJN2003849 12720578

[fsn31164-bib-0017] Han, M. , Song, P. , Huang, C. , Rezaei, A. , Farrar, S. , Brown, M. A. , & Ma, X. (2016). Dietary grape seed proanthocyanidins (GSPs) improve weaned intestinal microbiota and mucosal barrier using a piglet model. Oncotarget, 7, 80313 10.18632/oncotarget.13450 27880936 PMC5348322

[fsn31164-bib-0018] Laere, K. M. J. V. , Hartemink, R. , Bosveld, M. , Schols, H. A. , & Voragen, A. G. J. (2000). Fermentation of plant cell wall derived polysaccharides and their corresponding oligosaccharides by intestinal bacteria. Journal of Agricultural & Food Chemistry, 48, 1644. 10820072 10.1021/jf990519i

[fsn31164-bib-0019] Lange, Y. , Echevarria, F. , & Steck, T. L. (1991). Movement of zymosterol, a precursor of cholesterol, among three membranes in human fibroblasts. Journal of Biological Chemistry, 266, 21439–21443. 1939176

[fsn31164-bib-0020] Lin, H. H. , Chen, J. H. , Huang, C. C. , & Wang, C. J. (2007). Apoptotic effect of 3,4‐dihydroxybenzoic acid on human gastric carcinoma cells involving JNK/p38 MAPK signaling activation. International Journal of Cancer, 120, 2306–2316. 10.1002/ijc.22571 17304508

[fsn31164-bib-0021] Liu, H. , Zhang, J. , Zhang, S. , Yang, F. , Thacker, P. A. , Zhang, G. , & Ma, X. (2014). Oral administration of Lactobacillus fermentum I5007 favors intestinal development and alters the intestinal microbiota in formula‐fed piglets. Journal of Agricultural & Food Chemistry, 62, 860. 24404892 10.1021/jf403288r

[fsn31164-bib-0022] Liu, J. B. , Xue, P. C. , Cao, S. C. , Liu, J. , Chen, L. , & Zhang, H. F. (2018). Effects of dietary phosphorus concentration and body weight on postileal phosphorus digestion in pigs. Animal Feed Science and Technology, 242, 86–94. 10.1016/j.anifeedsci.2018.06.003

[fsn31164-bib-0023] Liu, J. B. , Yan, H. L. , Zhang, Y. , Hu, Y. D. , & Zhang, H. F. (2019a). Effects of dietary energy and protein content and lipid source on growth performance and carcass traits in Pekin ducks. Poultry Science, pez217, 1‐9. 10.3382/ps/pez217 30995295

[fsn31164-bib-0024] Liu, J. B. , Zhang, Y. , Li, Y. , Yan, H. L. , & Zhang, H. F. (2019b). L‐tryptophan enhances intestinal integrity in diquat‐challenged piglets associated with improvement of redox status and mitochondrial function. Animals, 9, 266 10.3390/ani9050266 PMC6562546 31121956

[fsn31164-bib-0025] Ma, T. Y. , Hoa, N. , Akotia, V. , & Chen, J. (2002). Mesalamine prevents TNF‐a induced increase in intestinal epithelial tight junction permeability. American Journal of Gastroenterology, 97, S271–S272. 10.1016/S0002-9270(02)05309-1

[fsn31164-bib-0026] Malina, H. Z. , Richter, C. , Mehl, M. , & Hess, O. M. (2001). Pathological apoptosis by xanthurenic acid, a tryptophan metabolite: Activation of cell caspases but not cytoskeleton breakdown. BMC Physiology, 1, 7 10.1186/1472-6793-1-7 11459518 PMC34606

[fsn31164-bib-0027] Malo, M. S. , Alam, S. N. , Mostafa, G. , Zeller, S. J. , Johnson, P. V. , Mohammad, N. , … Hodin, R. A. (2010). Intestinal alkaline phosphatase preserves the normal homeostasis of gut microbiota. Gut, 59, 1476–1484. 10.1136/gut.2010.211706 20947883

[fsn31164-bib-0028] Martin, F. P. J. , Sprenger, N. , Montoliu, I. , Rezzi, S. , Kochhar, S. , & Nicholson, J. K. (2010). Dietary modulation of gut functional ecology studied by fecal metabonomics. Journal of Proteome Research, 9, 5284–5295. 10.1021/pr100554m 20806900

[fsn31164-bib-0029] Mccann, M. J. , Zaneta, P. , Mcnabb, W. C. , Cookson, A. L. , Anderson, R. C. , Kelly, W. J. , & Roy, N. C. (2010). Lactobacillus plantarum MB452 enhances the function of the intestinal barrier by increasing the expression levels of genes involved in tight junction formation. BMC Microbiology, 10, 316. 21143932 10.1186/1471-2180-10-316 PMC3004893

[fsn31164-bib-0030] Mitsuharu, M. , Ryoko, K. , Takushi, O. , Yuji, A. , Shin, K. , Emiko, S. , & Yoshimi, B. (2012). Impact of intestinal microbiota on intestinal luminal metabolome. Scientific Reports, 2, 233. 22724057 10.1038/srep00233 PMC3380406

[fsn31164-bib-0031] Moreira, A. P. , Texeira, T. F. , Ferreira, A. B. , Peluzio, M. C. , & Alfenas, R. C. (2012). Influence of a high‐fat diet on gut microbiota, intestinal permeability and metabolic endotoxaemia. British Journal of Nutrition, 108, 801–809. 10.1017/S0007114512001213 22717075

[fsn31164-bib-0032] Neyrinck, A. M. , Possemiers, S. , Druart, C. , Van de Wiele, T. , De Backer, F. , Cani, P. D. , … Delzenne, N. M. (2011). Prebiotic effects of wheat arabinoxylan related to the increase in bifidobacteria, Roseburia and Bacteroides/Prevotella in diet‐induced obese mice. PLoS ONE, 6, e20944 10.1371/journal.pone.0020944 21695273 PMC3111466

[fsn31164-bib-0033] Neyrinck, A. M. , Van Hee, V. F. , Piront, N. , De Backer, F. , Toussaint, O. , Cani, P. D. , & Delzenne, N. M. (2012). Wheat‐derived arabinoxylan oligosaccharides with prebiotic effect increase satietogenic gut peptides and reduce metabolic endotoxemia in diet‐induced obese mice. Nutrition and Diabetes, 2, e28 10.1038/nutd.2011.24 23154683 PMC3302144

[fsn31164-bib-0034] Parikh, R. M. , Joshi, S. R. , Menon, P. S. , & Shah, N. S. (2010). Prevalence and pattern of diabetic dyslipidemia in Indian type 2 diabetic patients. Diabetes & Metabolic Syndrome: Clinical Research & Reviews, 4, 10–12. 10.1016/j.dsx.2009.04.005

[fsn31164-bib-0035] Polakof, S. , Díaz‐Rubio, M. E. , Dardevet, D. , Martin, J.‐F. , Pujos‐Guillot, E. , Scalbert, A. , … Comte, B. (2013). Resistant starch intake partly restores metabolic and inflammatory alterations in the liver of high‐fat‐diet‐fed rats. Journal of Nutritional Biochemistry, 24, 1920–1930. 10.1016/j.jnutbio.2013.05.008 24011718

[fsn31164-bib-0036] Riccardi, G. , Giacco, R. , & Rivellese, A. A. (2004). Dietary fat, insulin sensitivity and the metabolic syndrome. Clinical Nutrition, 23, 447–456. 10.1016/j.clnu.2004.02.006 15297079

[fsn31164-bib-0037] Rowbury, R. J. , & Woods, D. D. (1964). O‐succinylhomoserine as an intermediate in the synthesis of cystathionine by *Escherichia coli* . Journal of General Microbiology, 36, 341 10.1099/00221287-36-3-341 14217349

[fsn31164-bib-0038] Salden, B. N. , Troost, F. J. , Wilms, E. , Truchado, P. , Vilchez‐Vargas, R. , Pieper, D. H. , … Masclee, A. A. (2017). Reinforcement of intestinal epithelial barrier by arabinoxylans in overweight and obese subjects: A randomized controlled trial: Arabinoxylans in gut barrier. Clinical Nutrition, 37, 471–480. 10.1016/j.clnu.2017.01.024 28214040

[fsn31164-bib-0039] Turner, P. C. , Wu, Q. K. , Piekkola, S. , Gratz, S. , Mykkänen, H. , & El‐Nezami, H. (2008). *Lactobacillus rhamnosus* strain GG restores alkaline phosphatase activity in differentiating Caco‐2 cells dosed with the potent mycotoxin deoxynivalenol. Food & Chemical Toxicology an International Journal Published for the British Industrial Biological Research Association, 46, 2118 10.1016/j.fct.2008.02.004 18343010

[fsn31164-bib-0040] Ulluwishewa, D. , Anderson, R. C. , Mcnabb, W. C. , Moughan, P. J. , Wells, J. M. , & Roy, N. C. (2011). Regulation of tight junction permeability by intestinal bacteria and dietary components. Journal of Nutrition, 141, 769–776. 10.3945/jn.110.135657 21430248

[fsn31164-bib-0041] Van den Abbeele, P. , Gérard, P. , Rabot, S. , Bruneau, A. , El Aidy, S. , Derrien, M. , … Possemiers, S. (2011). Arabinoxylans and inulin differentially modulate the mucosal and luminal gut microbiota and mucin‐degradation in humanized rats. Environmental Microbiology, 13, 2667–2680. 10.1111/j.1462-2920.2011.02533.x 21883787

[fsn31164-bib-0042] Wang, T. J. , Ngo, D. , Psychogios, N. , Dejam, A. , Larson, M. G. , Vasan, R. S. , … Gerszten, R. E. (2013). 2‐Aminoadipic acid is a biomarker for diabetes risk. Journal of Clinical Investigation, 123, 4309 10.1172/JCI64801 24091325 PMC3784523

[fsn31164-bib-0043] William, H. (1995). Association of Official Analytical Chemists Official Methods of Analysis (AOAC), (13th ed). Washington DC: Academy Press, 34.

[fsn31164-bib-0044] Yan, H. L. , Zhang, L. , Guo, Z. D. , Zhang, H. F. , & Liu, J. B. (2019). Production phase affects the bioaerosol microbial composition and functional potential in swine confinement buildings. Animals, 9, 90. 10.3390/ani9030090 PMC6466638 30871116

[fsn31164-bib-0045] Zhang, Y. , Yu, B. , Yu, J. , Zheng, P. , Huang, Z. Q. , Luo, Y. H. , … & Chen, D. W. (2019). Butyrate promotes slow-twitch myofiber formation and mitochondrial biogenesis in finishing pigs via inducing specific microRNAs and PGC-1α expression. Journal of Animal Science, 97, 3180–3192. 10.1093/jas/skz187 31228349 PMC6667260

[fsn31164-bib-0046] Zhou, X. , He, L. , Wu, C. , Zhang, Y. , Wu, X. , & Yin, Y. (2017). Serine alleviates oxidative stress via supporting glutathione synthesis and methionine cycle in mice. Molecular Nutrition & Food Research, 61, 488‐498. 10.1002/mnfr.201700262 28759161

