# Supplementary material for: Effect of arabinoxylan on colonic bacterial metabolites and mucosal barrier in high‐fat diet‐induced rats
Source: Food Sci Nutr. 2019 Aug 12;7(9):3052–61. doi: 10.1002/fsn3.1164 (PMC6766541; doi:10.1002/fsn3.1164)
Supplement: Supplementary file 1 [file FSN3-7-3052-s001.docx]

**Supplementary material**

**Supplementary Table 1. Composition of experimental diets (as-fed basis)**

| Ingredient, *%* | HFD | AXD |
| --- | --- | --- |
| Corn starch | 45 | 42.3 |
| Defatted soybean powder | 23 | 21.6 |
| Wheat starch | 10 | 9.4 |
| Fish meal | 2 | 1.88 |
| Lard | 15 | 14.1 |
| Corn bran | 2 | 1.88 |
| Arabinoxylan | -- | 6 |
| Sodium chloride | 0.2 | 0.19 |
| Lysine-HCl | 0.12 | 0.11 |
| D,L-Methionine | 0.13 | 0.13 |
| Choline chloride | 0.1 | 0.1 |
| Surcose | 0.1 | 0.1 |
| Monocalcium phosphate | 1 | 0.94 |
| Limestone | 1.3 | 1.22 |
| Trace premix | 0.03 | 0.03 |
| Vitamin premix | 0.02 | 0.02 |
| Total | 100 | 100 |
| HFD: high-fat diet, AXD: high-fat supplemented with arabinoxylan (94% HF diet and 6% AX) | | |

**Supplementary table 2 The primers of colonic genes for RT-PCR (5′to 3′)**

| Primers |  | Nucleotide sequence |
| --- | --- | --- |
| β-actin | Forward | ACTATCGGCAATGAGCGGTTCC |
|  | Reverse | CTGTGTTGGCATAGAGGTCTTTACG |
| GAPDH | Forward | TCATCTCTGCCCCCTCTGCT |
|  | Reverse | CGACGCCTGCTTCACCACCT |
| TLR2 | Forward | TATCAGTCCCAAAGTCTAAAGTCG |
|  | Reverse | CTACCTCCGACAGTTCCAAGATG |
| TLR4 | Forward | GCCGGAAAGTTATTGTGGTGGT |
|  | Reverse | ATGGGTTTTAGGCGCAGAGTTT |
| NF-κB | Forward | GCGCATCCAGACCCCTCA |
|  | Reverse | TGGTATCTGTGCTTCTCTCT |
| MyD88 | Forward | AAAGGAACTGGGAGGCATCA |
|  | Reverse | CTGTTCTAGTTGCCGGATCAT |
| ZO-1 | Forward | ACCCGAAACTGATGCTGTGGATAG |
|  | Reverse | AAATGGCCGGGCAGAACTTGTGTA |
| Occludin | Forward | ATGTCCGGCCGATGCTCTC |
|  | Reverse | TTTGGCTGCTCTTGGGTCTGTAT |
| Claudin-1 | Forward | AGGAAAGGCCCTTCAGCAGAGCAA |
|  | Reverse | GTGCCCCCTCTTGACTCATGCAAC |
| Bcl-2 | Forward | ACGGTGGTGGAGGAACTCTTC |
|  | Reverse | ACACATGACCCCACCGAACT |
| Bax | Forward | TGCTACAGGGTTTCATCCAG |
|  | Reverse | ATCCACATCAGCAATCATCC |
| GAPDH: glyceraldehyde-3-phosphate dehydrogenase; ADRP: adipose differentiation-related protein; ZO-1: zonula occludens-1; Bcl2, B-cell lymphoma/leukaemia-2; Bax, Bcl-2-associated X protein. | | |

**Supplementary table 3 The primers of intestinal bacteria for RT-PCR (5′to 3′)**

| Primers |  | Nucleotide sequence |
| --- | --- | --- |
| *Escherichia coli* | Forward | CATGCCGCGTGTATGAAGAA |
|  | Reverse | CGGGTAACGTCAATGAGCAAA |
| *Bifidobacterium* | Forward | CGCGTCYGGTGTGAAAG |
|  | Reverse | CCCCACATCCAGCATCCA |
| *Lactobacillus* | Forward | GAGGCAGCAGTAGGGAATCTTC |
|  | Reverse | GGCCAGTTACTACCTCTATCCTTCTTC |
| *Bacteroidetes* | Forward | GGAGYATGTGGTTTAATTCGAAGCA |
|  | Reverse | AGCTGACGACAACCATGCAC |
|  | | |
